# Bioinformatic screening for key miRNAs and genes associated with myocardial infarction

**DOI:** 10.1002/2211-5463.12423

**Published:** 2018-04-19

**Authors:** Ke Wu, Qiang Zhao, Zhengmei Li, Nannan Li, Qiang Xiao, Xiuchang Li, Quanming Zhao

**Affiliations:** ^1^ Department of Cardiology Beijing Anzhen Hospital Capital Medical University Beijing China; ^2^ Department of Cardiology Central Hospital of Taian of Shandong Province China; ^3^ Department of Cardiology Affiliated Hospital of Taishan Medical University of Shandong Province Taian China; ^4^ Department of Radiology Taishan Medical University of Shandong Province Taian China; ^5^ Department of Respiration Medicine Central Hospital of Taian of Shandong Province China

**Keywords:** diagnostic biomarkers, miRNA‐target network, myocardial infarction, protein–protein interaction network

## Abstract

Despite significant advances in understanding of the causes of and treatment of myocardial infarction (MI) in recent years, morbidity and mortality is still high. The aim of this study was to identify miRNA and genes potentially associated with MI. mRNA and miRNA expression datasets were downloaded from the Gene Expression Omnibus database (http://www.ncbi.nlm.nih.gov/geo/). Interactions between miRNA and the expression and function of target genes were analyzed, and a protein–protein interaction network was constructed. The diagnostic value of identified miRNA and genes was assessed. Quantitative RT‐PCR was applied to validate the results of the bioinformatics analysis. MiR‐27a, miR‐31*, miR‐1291, miR‐139‐5p, miR‐204, miR‐375, and target genes including *CX3CR1*,*HSPA6,* and *TPM3* had potential diagnostic value. The genes *TFEB*,*IRS2*,*GRB2*,*FASLG*,*LIMS1*,*CX3CR1*,*HSPA6*,*TPM3*,*LAT2*,*CEBPD*,*AQP9,* and *MAPKAPK2* were associated with recovery from MI. In conclusion, the identified miRNA and genes might be associated with the pathology of MI.

**Abbreviations**
AQP9: aquaporin 9
AUC: area under the curve
CEBPD: CCAAT/enhancer binding protein delta
CX3CR1: C‐X3‐C motif chemokine receptor 1
DEGs: differentially expressed genes
FASLG: fas ligand
FDR: false discovery rate
GEO: gene expression omnibus
GO: gene ontology
GRB2: growth factor receptor bound protein 2
HCM: hypertrophic cardiomyopathy
HSPA6: heat shock protein family A (Hsp70) member 6
IRS2: insulin receptor substrate 2
KEGG: kyoto encyclopedia of genes genomes
LAT2: linker for activation of T cells family member 2
LIMS1: LIM zinc finger domain containing 1
MAPKAPK2: mitogen‐activated protein kinase‐activated protein kinase 2
MI: myocardial infarction
PPI: protein–protein interaction
qRT‐PCR: quantitative RT‐PCR
ROC: receiver operating characteristic
TFEB: transcription factor EB
TPM3: tropomyosin 3

Myocardial infarction (MI) is the multifactorial injurious event, which involves all the components of the cardiac myocyte 1. The partial or complete occlusion of a coronary artery is the cause of MI, which leads to apoptosis and necrosis in the myocardium. It is reported that smoking, hypertension, diabetes mellitus, hypercholesterolemia, or dyslipidemia are the main causal factors of MI 2. Atherosclerosis also leads to MI 3. Even though survival rates after MI have been remarkably improved by early revascularization therapy and drug treatment, a significant number of patients develop heart failure 4. Despite significant advances in understanding of the causes of and treatment of MI in recent years, morbidity and mortality is still high 5.

miRNA are small, noncoding RNA that regulate the expression of target genes at the post‐transcription level. miRNA can modulate important complex gene regulatory pathways involved in cardiovascular development 6, 7, 8. More and more evidence reveals that signature expression pattern of miRNA plays a vital role in MI, cardiac arrhythmia, and pathological cardiac hypertrophy 9. It is noted that some miRNA expressed in heart are remarkably deregulated in patients with acute MI compared with healthy controls 10, 11. It is found that several miRNA including miR‐1, miR‐21, miR‐206, and miR‐499‐5p are deregulated in MI 12, 13, 14. Clinically, myoglobin, cardiac troponins, N‐terminal probrain natriuretic peptide, creatine kinases, and lactate dehydrogenase have been considered as diagnosis biomarkers of patients with acute MI 15, 16, 17, 18. It is worth mentioning that several miRNA including miR‐1, miR‐208α, miR‐126, miR‐122‐5p, and miR‐19a have been recognized as novel biomarkers for early diagnosis of acute MI 19, 20, 21, 22. Therefore, improving knowledge about the interaction between miRNA and target genes may be helpful in finding new pathological mechanism and markers for MI.

In this study, we aimed to find differentially expressed miRNA and genes in MI by integrated analysis. The miRNA‐gene target analysis was subsequently performed. Then, functional enrichment analysis including Gene Ontology (GO) and Kyoto Encyclopedia of Genes Genomes (KEGG) was used to investigate the biological function of genes followed by construction of a protein–protein interaction (PPI) network of top 100 differentially expressed genes (DEGs; 50 up‐regulated and 50 down‐regulated). Receiver operating characteristic (ROC) analysis was applied to analyze the diagnostic usefulness of identified differentially expressed miRNA and genes. Quantitative RT‐PCR (qRT‐PCR) was used to validate the result of the bioinformatics analysis. http://www.ncbi.nlm.nih.gov/geo/query/acc.cgi?acc=GSE29532 and http://www.ncbi.nlm.nih.gov/geo/query/acc.cgi?acc=GSE48060 datasets were used for expression and recovery analysis of DEGs. Our study may be helpful in understanding the pathogenic mechanism and finding valuable diagnosis biomarkers for MI.

## Materials and methods

### Datasets

In this study, we searched datasets from the Gene Expression Omnibus (GEO) database (http://www.ncbi.nlm.nih.gov/geo/). The study type was characterized as ‘expression profiling by array’. All selected datasets were genome‐wide expression data of mRNA/miRNA from MI group and normal group blood samples. And those standardized or primary datasets were included in this study. Finally, a total of three mRNA datasets (http://www.ncbi.nlm.nih.gov/geo/query/acc.cgi?acc=GSE34198, http://www.ncbi.nlm.nih.gov/geo/query/acc.cgi?acc=GSE48060, and http://www.ncbi.nlm.nih.gov/geo/query/acc.cgi?acc=GSE61145) and two miRNA datasets (http://www.ncbi.nlm.nih.gov/geo/query/acc.cgi?acc=GSE31568 and http://www.ncbi.nlm.nih.gov/geo/query/acc.cgi?acc=GSE61741) were screened, which was shown in Table 1.

**Table 1:** The mRNA and miRNA datasets

GEO accession		Platform	Samples (N : P)	Country
http://www.ncbi.nlm.nih.gov/geo/query/acc.cgi?acc=GSE34198	mRNA‐array	GPL6102 Illumina human‐6 v2.0 expression beadchip	48 : 45	Czech Republic
http://www.ncbi.nlm.nih.gov/geo/query/acc.cgi?acc=GSE48060	mRNA‐array	GPL570 [HG‐U133_Plus_2] Affymetrix Human Genome U133 Plus 2.0 Array	21 : 31	USA
http://www.ncbi.nlm.nih.gov/geo/query/acc.cgi?acc=GSE61145	mRNA‐array	GPL6106 Sentrix Human‐6 v2 Expression BeadChip	10 : 7	South Korea
http://www.ncbi.nlm.nih.gov/geo/query/acc.cgi?acc=GSE31568	miRNA‐array	GPL9040 febit Homo Sapiens miRBase 13.0	70 : 20	Germany
http://www.ncbi.nlm.nih.gov/geo/query/acc.cgi?acc=GSE61741	miRNA‐array	GPL9040 febit Homo Sapiens miRBase 13.0	94 : 62	Germany

N, normal; P, patients.

### Identification of differentially expressed genes and differentially expressed miRNA

With numbers of publicly available microarray databases, there will be an interest in combining data from different platforms. It is noted that metaMA package allows this integration of different platforms as it can handle missing data and eliminate the batch effects 23. In this study, Limma and metaMA packages were used to identify the DEGs. The normal inverse method was used to combine the *P* value in metaMA. The false discovery rate (FDR) was performed for multiple testing corrections of raw *P* value through the Benjamin and Hochberg method 24, 25. The threshold of DEGs was set as FDR < 0.05. FDR < 0.05 & |Combined.ES|> 0.8 was the threshold of identifying differentially expressed miRNA.

### miRNA‐gene target analyses

Identifying the target genes of miRNA is a crucial step in exploring the function of miRNA in specific tissues and cells. Herein, six miRNA‐target prediction tools (RNA22, miRanda, miRDB, miRWalk, PICTAR2, and Targetscan) were applied to predict the target DEGs of differentially expressed miRNA. The miRNA‐targets that were predicted by more than four algorithms or verified by experiment in miRWalk database were screened out. Then, the miRNA‐target regulatory network was constructed, which was visualized using Cytoscape Software 26.

### Functional annotation analyses of miRNA‐target differentially expressed genes

To obtain the biological function and signaling pathways of miRNA‐target DEGs, the GeneCoDis3 (http://genecodis.cnb.csic.es/analysis) software was used for GO (http://www.geneontology.org/) annotation and KEGG (http://www.genome. jp/kegg/pathway.html) pathway enrichment analysis. The threshold of GO function and KEGG pathway of DEGs was all set as FDR < 0.05.

### Protein–protein interaction network construction

It is useful for understanding the molecule mechanism of MI by studying the interactions between proteins. To gain insights into the interaction between proteins encoded by DEGs and other proteins, the database of BioGRID (http://thebiogrid.org) was used to retrieve the predicted interactions between top 100 proteins encoded by DEGs (50 up‐regulated and 50 down‐regulated) and other proteins. Then, PPI network was visualized by the Cytoscape Software (http://cytoscape.org/). A node in the PPI network denotes protein, and the edge denotes the interactions.

### Receiver operating characteristic analyses

Using pROC package in R language, we performed the receiver operating characteristic analyses to assess the diagnostic value of DEGs. The area under the curve (AUC) under binomial exact confidence interval was calculated, and the receiver operating characteristic curve was generated.

### Validation of quantitative RT‐PCR

In this study, five patients diagnosed as MI and five normal individuals were enrolled in this study. Both MI and corresponding normal blood samples were obtained and immediately frozen in liquid nitrogen. All participating individuals provided informed consent with the approval of the ethics committee of our hospital.

Total RNA of fresh blood samples from MI patients and normal individuals was extracted using TRizol reagent (Invitrogen, Foster City, CA, USA) according to the manual instructions. SuperScript III Reverse Transcription Kit (Invitrogen) was used to synthesize the cDNA. qRT‐PCR was performed using SYBR Green PCR Master Mix (Applied Biosystems, Foster City, CA, USA) on Applied Biosystems 7500 (Applied Biosystems). GAPDH served as internal control for gene detection, and the relative expression of genes was calculated using the fold change equation.

### Expression analyses in the early stage of myocardial infarction and recovery‐related analysis of differentially expressed genes

To analyze the expression of DEGs in the early stage (different blood collection time points, time 1, time 2, time 3, time 4, time 5, and time 6) of MI in the dataset of http://www.ncbi.nlm.nih.gov/geo/query/acc.cgi?acc=GSE29532 and further investigate the association between DEGs and MI recovery in the dataset of http://www.ncbi.nlm.nih.gov/geo/query/acc.cgi?acc=GSE48060, 12 DEGs including transcription factor EB (TFEB), insulin receptor substrate 2 (IRS2), growth factor receptor bound protein 2 (GRB2), fas ligand (FASLG), LIM zinc finger domain containing 1 (LIMS1), C‐X3‐C motif chemokine receptor 1 (CX3CR1), heat shock protein family A (Hsp70) member 6 (HSPA6), tropomyosin 3 (TPM3), linker for activation of T cells family member 2 (LAT2), CCAAT/enhancer binding protein delta (CEBPD), aquaporin 9 (AQP9), and mitogen‐activated protein kinase‐activated protein kinase 2 (MAPKAPK2) were selected for analysis.

## Results

### Differentially expressed genes and differentially expressed miRNA analysis

**Figure 1:**
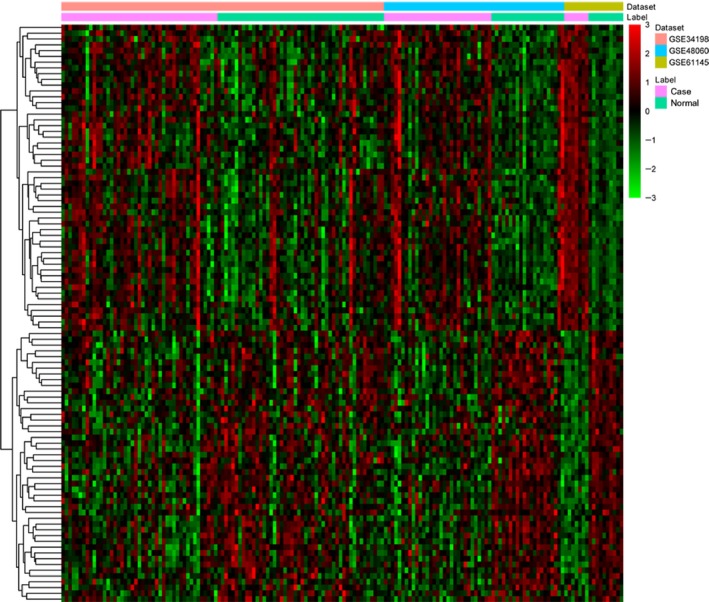
Heat map of top 100 DEGs in MI. Diagram presents the result of a two‐way hierarchical clustering of top 100 DEGs and samples. The clustering is constructed using the complete‐linkage method together with the Euclidean distance. Each row represents a DEG and each column, a sample. DEGs clustering tree is shown on the right. The color scale illustrates the relative level of DEGs expression: purple, below the reference channel; green, higher than the reference.

**Figure 2:**
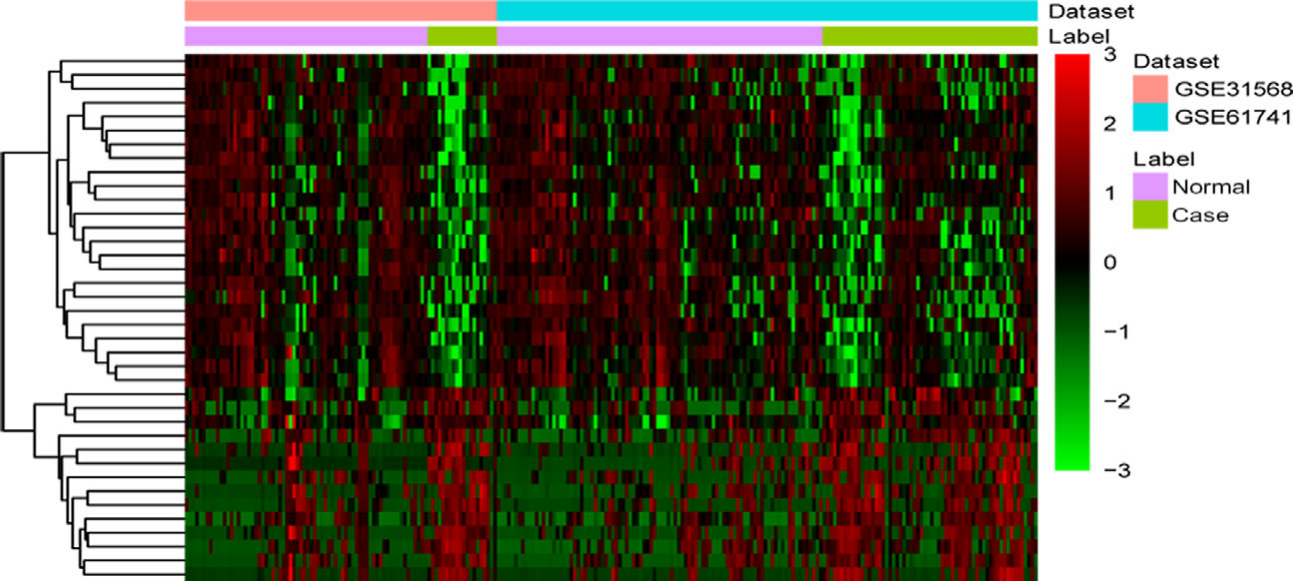
Heat map of all differentially expressed miRNA in MI. Diagram presents the result of a two‐way hierarchical clustering of all differentially expressed miRNA and samples. The clustering is constructed using the complete‐linkage method together with the Euclidean distance. Each row represents a miRNA and each column, a sample. The miRNA clustering tree is shown on the right. The color scale illustrates the relative level of miRNA expression: purple, below the reference channel; green, higher than the reference.

A total of 1007 DEGs were identified as the threshold of FDR < 0.05, consisting of 564 up‐regulated and 443 down‐regulated genes. Top 10 up‐ and down‐regulated DEGs were presented in Table 2. The heat map of top 100 DEGs was shown in Fig. 1. All DEGs were listed in the Table S1. In addition, a total of 38 differentially expressed miRNA including 14 up‐regulated and 24 down‐regulated miRNA. Top 10 up‐ and down‐regulated differentially expressed miRNA was listed in Table 3. Figure 2 showed the heat map of all differentially expressed miRNA.

**Table 2:** Top 10 up‐ and down‐regulated DEGs

ID	Symbol	Combined.ES	*P* value	FDR	Up/Down
55350	VNN3	1.098788106	1.68E‐08	0.00015739	Up
1912	PHC2	1.106042072	4.44E‐08	0.000207716	Up
7942	TFEB	0.930874415	8.63E‐08	0.000269206	Up
8291	DYSF	0.944578311	2.10E‐07	0.000393166	Up
7462	LAT2	0.91656781	1.75E‐07	0.000393166	Up
2137	EXTL3	0.978263843	9.75E‐07	0.000707473	Up
1052	CEBPD	0.93852956	1.03E‐06	0.000707473	Up
366	AQP9	0.920634347	7.99E‐07	0.000707473	Up
89846	FGD3	0.86933981	1.06E‐06	0.000707473	Up
10043	TOM1	0.811419165	1.01E‐06	0.000707473	Up
54438	GFOD1	−0.857872809	2.85E‐07	0.000444736	Down
81537	SGPP1	−0.925693388	4.02E‐07	0.000538047	Down
356	FASLG	−0.898564987	4.97E‐07	0.000544713	Down
130814	PQLC3	−0.888672951	5.24E‐07	0.000544713	Down
22836	RHOBTB3	−0.763444842	3.05E‐06	0.001188091	Down
3560	IL2RB	−0.890989888	3.60E‐06	0.001227364	Down
1524	CX3CR1	−0.854049867	4.19E‐06	0.00126511	Down
9788	MTSS1	−0.818619651	4.16E‐06	0.00126511	Down
962	CD48	−0.925369052	4.83E‐06	0.001414235	Down
51699	VPS29	−0.772616109	5.02E‐06	0.001422705	Down

**Table 3:** Top 10 up‐ and down‐regulated differentially expressed miRNA

Symbol	Combined.ES	*P* value	FDR	Up/Down
hsa‐miR‐375	0.964233114	3.81E‐11	3.23E‐08	Up
hsa‐miR‐520c‐3p	0.928272928	2.90E‐10	8.18E‐08	Up
hsa‐miR‐132*	0.897286366	4.05E‐10	8.58E‐08	Up
hsa‐miR‐204	0.906784899	5.45E‐10	9.24E‐08	Up
hsa‐miR‐142‐3p	0.87693145	1.32E‐09	1.86E‐07	Up
hsa‐miR‐29c*	0.83581277	3.13E‐09	3.31E‐07	Up
hsa‐miR‐1274b	0.803505509	1.26E‐08	9.72E‐07	Up
hsa‐miR‐1258	1.030135467	1.23E‐07	6.49E‐06	Up
hsa‐miR‐1468	0.965203705	5.28E‐05	0.000520325	Up
hsa‐miR‐609	0.887322197	0.000406945	0.00271117	Up
hsa‐miR‐200a	−0.927575354	2.63E‐10	8.18E‐08	Down
hsa‐miR‐767‐5p	−0.871863499	1.94E‐09	2.35E‐07	Down
hsa‐miR‐455‐3p	−0.838268201	7.37E‐09	6.94E‐07	Down
hsa‐miR‐646	−0.836200084	8.52E‐09	7.22E‐07	Down
hsa‐miR‐627	−0.800880075	3.77E‐08	2.46E‐06	Down
hsa‐miR‐1245	−0.917431476	4.02E‐07	1.46E‐05	Down
hsa‐miR‐515‐5p	−1.095131558	1.33E‐05	0.000176153	Down
hsa‐miR‐519b‐5p	−0.893325033	1.36E‐05	0.000177605	Down
hsa‐miR‐155*	−0.921431507	7.49E‐05	0.000694016	Down
hsa‐miR‐330‐3p	−0.968066113	0.000129252	0.001085202	Down

### miRNA‐target gene interactions

**Figure 3:**
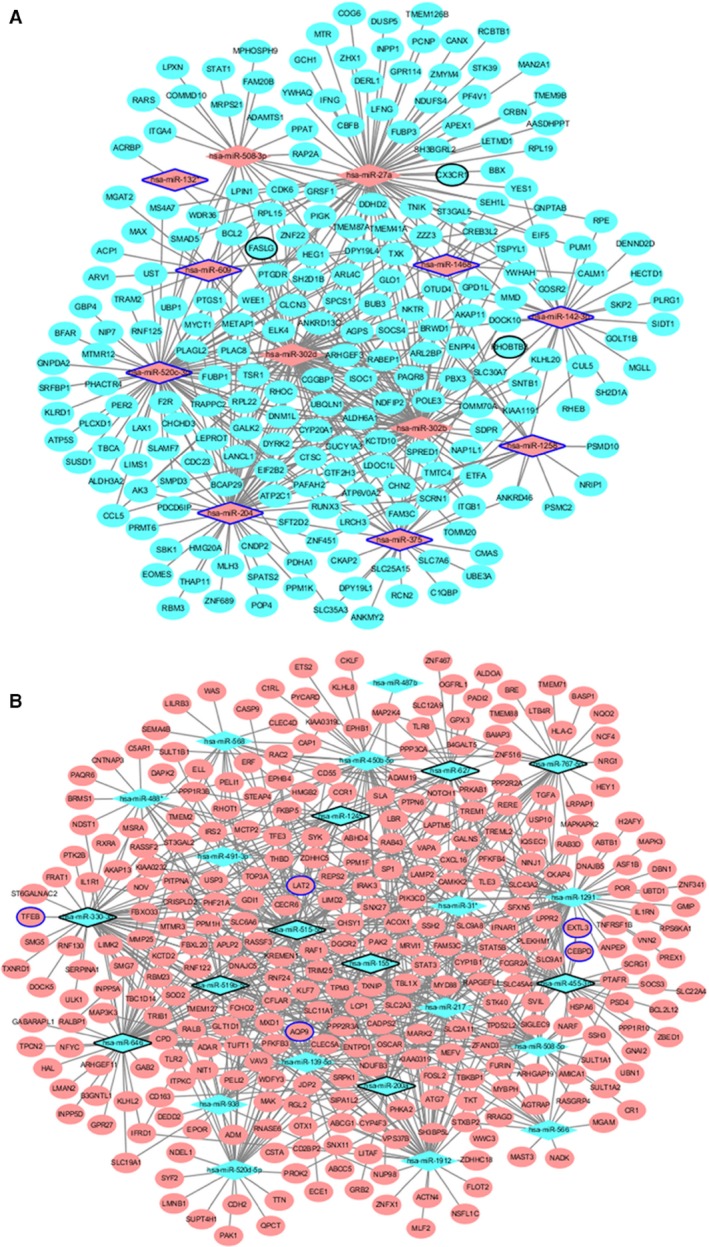
Interaction networks of miRNA and target DEGs in MI. (A) Up‐regulated miRNA and target genes; (B) the down‐regulated miRNA and target genes. The rhombus and ellipses represent the miRNA and target DEGs, respectively. The red and green colors represent up‐regulation and down‐regulation, respectively. Ellipses with blue and black border represent top 10 up‐regulation and down‐regulation, respectively.

A total of 1186 miRNA‐target pairs including 392 up‐regulated miRNA‐down‐regulated target pairs and 639 down‐regulated miRNA‐up‐regulated target pairs were obtained. Among which, 132 up‐regulated miRNA‐down‐regulated target pairs and 113 down‐regulated miRNA‐up‐regulated target pairs have been confirmed by miRWalk. The MI‐specific miRNA‐target interaction network was shown in Fig. 3. Table 4 listed the target DEGs of differentially expressed miRNA.

**Table 4:** The target DEGs of differentially expressed miRNA

miRNA	Up/Down	Count	Target mRNA
hsa‐miR‐302d	Up	75	PIGK, DNM1L, CDK6, LANCL1, FAM3C, AKAP11, CHN2, SH2D1B, SOCS4, SLC30A7, SPRED1, ELK4, ETFA, ENPP4, TNIK, METAP1, ATP6V0A2, GALK2, ZZZ3, ATP2C1, GLO1, DPY19L4, GUCY1A3, UBQLN1, FASLG, ITGB1, SMAD5, NAP1L1, NKTR, ARHGEF3, AK3, PBX3, PLAC8, PLAGL2, BRWD1, POLE3, NDFIP2, OTUD4, BCAP29, PTGDR, CYP20A1, PTGS1, HEG1, RPL15, RPL22, TRAPPC2, CREB3L2, SNTB1, TSPYL1, WEE1, ANKRD13C, KCTD10, LDOC1L, SDPR, DYRK2, PAQR8, AGPS, CGGBP1, RUNX3, ST3GAL5, FUBP1, RABEP1, TOMM20, TOMM70A, CLCN3, SPCS1, GTF2H3, RHOC, ISOC1, TSR1, CCL5, TXK, EIF2B2, BUB3, SCRN1
hsa‐miR‐27a	Up	71	PIGK, ARL4C, CDK6, YWHAQ, ZHX1, CLCN3, SOCS4, WDR36, SLC30A7, CX3CR1, DUSP5, EIF5, GPR114, TNIK, LPIN1, DDHD2, MMD, ARL2BP, LETMD1, TMEM87A, ZZZ3, GCH1, STK39, DPY19L4, GRSF1, APEX1, INPP1, SMAD5, MAN2A1, MTR, NDUFS4, NKTR, PAFAH2, CRBN, PF4V1, PLAGL2, BRWD1, PPAT, RCBTB1, TMEM126B, TMEM9B, BBX, PCNP, PTGDR, HEG1, COG6, MS4A7, RAP2A, BCL2, CREB3L2, TSPYL1, WEE1, YES1, ZNF22, SEH1L, CANX, SH3BGRL2, TMTC4, AGPS, CBFB, ST3GAL5, FUBP3, BUB3, ZMYM4, GOSR2, IFNG, LFNG, OTUD4, AASDHPPT, RPL19, DERL1
hsa‐miR‐520c‐3p	Up	70	DNM1L, UST, ARL4C, CDK6, GBP4, GNPDA2, WDR36, SLC30A7, SRFBP1, SPRED1, ELK4, F2R, ALDH3A2, METAP1, LPIN1, SLC35A3, GALK2, ATP5S, GLO1, DPY19L4, GTF2H3, GUCY1A3, FASLG, KLRD1, RHOC, LIMS1, SMAD5, PAFAH2, ARHGEF3, PBX3, BFAR, PLAC8, NIP7, PLAGL2, BRWD1, MTMR12, LEPROT, LAX1, CHCHD3, RNF125, PLCXD1, SMPD3, TSR1, BCAP29, KIAA1191, PTGDR, CYP20A1, PTGS1, HEG1, SLAMF7, MS4A7, CCL5, TRAPPC2, SUSD1, PHACTR4, TBCA, WEE1, MYCT1, LRCH3, AGPS, RUNX3, CDC23, PER2, TMEM41A, RABEP1, TRAM2, CLCN3, SPCS1, TXK, EIF2B2
hsa‐miR‐302b	Up	69	DNM1L, CDK6, LANCL1, AKAP11, CHN2, SH2D1B, SOCS4, SLC30A7, SPRED1, ELK4, ETFA, ENPP4, TNIK, GPD1L, METAP1, ATP6V0A2, GALK2, ATP2C1, KLHL20, GUCY1A3, UBQLN1, FASLG, ITGB1, NAP1L1, NKTR, ARHGEF3, AK3, PBX3, PLAC8, PLAGL2, BRWD1, POLE3, NDFIP2, OTUD4, DOCK10, BCAP29, PTGDR, CYP20A1, RPL15, RPL22, TRAPPC2, CREB3L2, SNTB1, TSPYL1, WEE1, ANKRD13C, KCTD10, LDOC1L, SDPR, DYRK2, PAQR8, AGPS, CGGBP1, RUNX3, ST3GAL5, RABEP1, TOMM70A, CLCN3, GLO1, SPCS1, GTF2H3, RHOC, ISOC1, TSR1, CCL5, TXK, EIF2B2, BUB3, SCRN1
hsa‐miR‐646	Down	61	TRIB1, ADAR, TXNIP, CAMKK2, RALBP1, LMAN2, RNF24, KLHL2, B3GNTL1, MARK2, TPCN2, KCTD2, GABARAPL1, FBXO33, RASSF3, HAL, NDST1, RAB43, IFRD1, IL1R1, INPP5A, INPP5D, MEFV, MAP3K3, MYD88, NFYC, NOV, ACOX1, RAPGEFL1, PFKFB3, PITPNA, TMEM127, TBC1D14, RAF1, RALB, ST3GAL2, SLC6A6, SLC9A1, SLC19A1, SOD2, SP1, STAT3, SVIL, SYK, TOP3A, TPM3, LAPTM5, KIAA0319L, DNAJC5, KREMEN1, KLF7, IRS2, MTMR3, ENTPD1, PPM1F, ARHGEF11, GAB2, SMG7, OSCAR, GPR27, SLC2A3
hsa‐miR‐1291	Down	59	MRVI1, SLC43A2, DBN1, MARK2, EXTL3, SLC9A8, DNAJB5, GALNS, CECR6, ANPEP, RAB43, IL1RN, MYBPH, NINJ1, NOTCH1, FURIN, PAK2, GMIP, PFKFB4, POR, ASF1B, MAPK3, PTAFR, PREX1, PTPN6, RAF1, RPS6KA1, ABHD4, LPPR2, SLC9A1, SLC11A1, SP1, STAT5B, SYK, TBL1X, TKT, TLE3, TNFRSF1B, TRIM25, LAPTM5, TREML2, UBTD1, ABTB1, LIMD2, STK40, KREMEN1, ZNF341, VNN2, REPS2, MAPKAPK2, SFXN5, ENTPD1, H2AFY, PPM1F, TBKBP1, PLEKHM1, IQSEC1, OSCAR, PFKFB3
hsa‐miR‐204	Up	54	PDCD6IP, DNM1L, ARL4C, LANCL1, HMG20A, CHN2, SOCS4, SLC30A7, PPM1K, SPRED1, ELK4, F2R, RHOBTB3, GPD1L, METAP1, DDHD2, TMEM87A, ZNF451, MLH3, SBK1, PAFAH2, AK3, BRWD1, PRMT6, SMPD3, CNDP2, BCAP29, THAP11, PTGDR, CYP20A1, PTGS1, SLAMF7, RBM3, BCL2, CREB3L2, SPATS2, UBP1, WEE1, ZNF22, MYCT1, EOMES, LDOC1L, PAQR8, AGPS, ST3GAL5, SCRN1, TOMM70A, POP4, ZNF689, SFT2D2, ALDH6A1, PDHA1, CHCHD3, CDC23
hsa‐miR‐330‐3p	Down	52	FRAT1, TRIB1, ST6GALNAC2, IRAK3, AKAP13, RNF24, FCHO2, JDP2, CPD, GLT1D1, PTK2B, WDFY3, SMG5, CECR6, AQP9, LAMP2, MXD1, MSRA, ACOX1, PFKFB3, SERPINA1, RNF130, PPM1H, TBC1D14, CXCL16, RRAGD, RAF1, RALB, RXRA, ABHD4, ST3GAL2, SOD2, TBL1X, TXNRD1, TRIM25, TFEB, PPP1R3B, STEAP4, DOCK5, DNAJC5, CRISPLD2, KREMEN1, ULK1, SSH2, MTMR3, VAPA, ENTPD1, ZNF516, KIAA0232, SMG7, RBM23, SP1
hsa‐miR‐200a	Down	46	CD2BP2, OSCAR, CPD, CSTA, FKBP5, WDFY3, GRB2, LBR, CYP4F3, MAK, MAP3K3, NDUFB3, NUP98, FURIN, ACOX1, PITPNA, PPP2R2A, PRKAB1, MCTP2, PPM1H, SIPA1L2, RALB, MAP2K4, SLC6A6, SP1, STAT5B, THBD, TOP3A, LAT2, DNAJC5, SH3BP5L, KLF7, IRS2, REPS2, CD163, ENTPD1, RAB3D, ZNF516, KIAA0319, FCHO2, CYP1B1, OTX1, RGL2, SOD2, STXBP2, RASSF2
hsa‐miR‐515‐5p	Down	46	IRAK3, EPHB4, ERF, CHSY1, FKBP5, FOSL2, TMEM2, ZDHHC5, GDI1, RASSF3, APLP2, IL1R1, RERE, PAK2, ACOX1, PHF21A, PITPNA, PPP2R2A, TMEM127, PPM1H, RAC2, RNASE6, SLC11A1, SP1, STAT5B, THBD, TPM3, TUFT1, PPP1R3B, LIMD2, ELL, CASP9, FBXL20, ARHGAP19, SSH2, KLF7, MTMR3, CADPS2, ENTPD1, RASSF2, USP3, DGCR2, VAV3, CD55, DAPK2, NOTCH1
hsa‐miR‐1912	Down	46	ADAR, MRVI1, ATG7, IRAK3, GLT1D1, FLOT2, SLC9A8, KCTD2, FOSL2, SNX11, RAB43, IFRD1, AQP9, MYBPH, MYD88, NUP98, RAPGEFL1, PFKFB3, TREM1, PRKAB1, TMEM127, MCTP2, NSFL1C, PELI2, ZNFX1, SLA, SLC2A3, SLC6A6, SLC11A1, SRPK1, STAT5B, SYK, TGFA, TKT, TPM3, MLF2, SH3BP5L, CFLAR, VAPA, LITAF, ENTPD1, RAB3D, ABCG1, TBKBP1, ACTN4, ZDHHC18
hsa‐miR‐508‐5p	Down	42	RASGRP4, AMICA1, JDP2, CR1, GLT1D1, CYP1B1, FKBP5, LAMP2, LCP1, MEFV, RERE, PAK2, ACOX1, PIK3CD, PPP2R3A, PTAFR, PROK2, ZFAND3, SLC11A1, SULT1A2, SULT1A1, TBL1X, TFE3, TPM3, TUFT1, LAT2, TRIM25, TREML2, ARHGAP19, CFLAR, SFXN5, DGCR2, OSCAR, NARF, SIGLEC9, HSPA6, SSH3, RBM23, FBXL20, SSH2, ENTPD1, RAB3D
hsa‐miR‐455‐3p	Down	40	RNF24, EXTL3, UBN1, LAMP2, RERE, PAK2, FAM53C, PFKFB4, PPP1R10, PPP2R2A, TMEM127, SLC45A4, PTAFR, SLC22A4, SRPK1, SVIL, TGFA, SNX27, KLF7, SOCS3, ZBED1, SFXN5, KIAA0319, CEBPD, TXNIP, CKAP4, SCRG1, FKBP5, PSD4, NARF, SIGLEC9, GNAI2, HSPA6, IFNAR1, ACOX1, CXCL16, TFE3, TPM3, BCL2L12, USP10
hsa‐miR‐450b‐5p	Down	40	MRVI1, CAP1, RALBP1, EPHB1, ETS2, CECR6, HMGB2, CLEC4D, RAB43, LAMP2, LBR, PAK2, CKLF, C1RL, PIK3CD, PPP2R2A, PPP3CA, PELI1, KLHL8, RAC2, ST3GAL2, SOD2, STAT5B, TPM3, C5AR1, STEAP4, KIAA0319L, SNX27, SSH2, KLF7, IRS2, ADAM19, USP10, VAPA, B4GALT5, ENTPD1, ZNF516, IQSEC1, SLC43A2, PYCARD
hsa‐miR‐520d‐5p	Down	33	CDH2, TRIB1, TXNIP, CAMKK2, FCHO2, WDFY3, QPCT, MXD1, MAK, PAK2, PHF21A, PFKFB3, PITPNA, RBM23, PRKAB1, RNASE6, SLC6A6, SRPK1, SUPT4H1, TBL1X, TTN, TUFT1, TRIM25, NDEL1, RAB3D, DGCR2, ADM, SYF2, LMNB1, PAK1, PROK2, SOD2, CRISPLD2
hsa‐miR‐139‐5p	Down	33	ABCC5, MRVI1, FCHO2, JDP2, OSCAR, ECE1, CLEC5A, APLP2, MXD1, NIT1, NOTCH1, PAK2, ACOX1, PITPNA, PPP2R3A, SLC45A4, ZFAND3, SRPK1, TBL1X, THBD, TPM3, TRIM25, VPS37B, ITPKC, DNAJC5, CRISPLD2, ARHGAP19, SSH2, KLF7, ABCG1, KIAA0319, HMGB2, RHOT1
hsa‐miR‐142‐3p	Up	30	MGLL, ANKRD46, SPRED1, RHOBTB3, MMD, HECTD1, KLHL20, DPY19L4, PLRG1, BRWD1, SIDT1, RHEB, RPE, DENND2D, CALM1, CUL5, GOSR2, PUM1, EIF5, GPD1L, SH2D1A, GOLT1B, OTUD4, DOCK10, KIAA1191, CREB3L2, SKP2, TSPYL1, YES1, YWHAH
hsa‐miR‐375	Up	29	ARL4C, ELK4, MMD, LEPROT, ANKMY2, SLC25A15, FAM3C, CTSC, ANKRD46, SPRED1, DPY19L1, SLC35A3, ARL2BP, CKAP2, ITGB1, SFT2D2, PAFAH2, CMAS, KIAA1191, CYP20A1, RCN2, BCL2, C1QBP, UBE3A, CUL5, KCTD10, TMTC4, PAQR8, SLC7A6
hsa‐miR‐767‐5p	Down	29	BASP1, CAMKK2, LTB4R, TMEM71, HEY1, NRG1, HLA‐C, MXD1, NCF4, NQO2, ACOX1, PIK3CD, PPP3CA, AGTRAP, MAP2K4, TGFA, TPM3, LAT2, SSH2, CFLAR, TMEM88, VAPA, B4GALT5, SFXN5, BRE, ZNF516, LRPAP1, TLE3, RAB3D
hsa‐miR‐627	Down	29	IRAK3, PADI2, ZNF467, GPX3, MYD88, RAPGEFL1, PFKFB4, PITPNA, SLC12A9, PTPN6, MAP2K4, SLA, TREML2, KREMEN1, MTMR3, BAIAP3, SFXN5, KIAA0232, CYP1B1, CD55, EPHB1, ALDOA, NINJ1, NOTCH1, TLR8, TREM1, PRKAB1, SP1, OGFRL1
hsa‐miR‐217	Down	28	ATG7, IRAK3, FCGR2A, CHSY1, LBR, MXD1, ACOX1, PHKA2, TREM1, WWC3, PPM1H, SLC6A6, SLC2A11, STAT5B, TGFA, TPD52L2, DNAJC5, ARHGAP19, CFLAR, MTMR3, CADPS2, ENTPD1, RAB3D, PPM1F, KIAA0232, PLEKHM1, CKAP4, STK40
hsa‐miR‐519b‐5p	Down	26	CAMKK2, IRAK3, FKBP5, WDFY3, SLC9A8, IFRD1, LAMP2, LCP1, LIMK2, PAK2, FAM53C, PHF21A, PIK3CD, PPP2R3A, PELI2, PTAFR, PPM1H, MAP2K4, MMP25, ST3GAL2, TLR2, RNF122, ARHGAP19, CFLAR, MTMR3, ENTPD1
hsa‐miR‐508‐3p	Up	22	ARL4C, MPHOSPH9, WDR36, ELK4, DPY19L4, ITGA4, COMMD10, PPAT, PTGS1, RAP2A, RARS, RPE, RPL15, STAT1, UBP1, YWHAH, MYCT1, LPXN, ADAMTS1, FAM20B, MRPS21, PUM1
hsa‐miR‐938	Down	19	TRIB1, ADAR, ADM, DEDD2, EPOR, CECR6, MXD1, NUP98, FAM53C, MAP2K4, LPPR2, SLC11A1, SLC19A1, SRPK1, DNAJC5, SSH2, ADAM19, CFLAR, VAV3
hsa‐miR‐568	Down	18	CAP1, SEMA4B, LILRB3, FBXO33, GALNS, CLEC4D, MSRA, NOTCH1, PHF21A, TREM1, RHOT1, MCTP2, SLC6A6, STAT5B, WAS, PPP1R3B, STEAP4, ELL
hsa‐miR‐488*	Down	17	AKAP13, NDST1, ACOX1, PHF21A, PELI1, PPM1H, SYK, TFE3, TPM3, C5AR1, CNTNAP3, PAQR6, SNX27, CFLAR, RASSF2, BRMS1, RXRA
hsa‐miR‐566	Down	17	IRAK3, RASGRP4, MAST3, MEFV, RAPGEFL1, AGTRAP, PTAFR, NADK, STAT3, TBL1X, TPM3, CRISPLD2, EXTL3, FOSL2, RRAGD, STXBP2, MGAM
hsa‐miR‐1258	Up	14	SLC30A7, ENPP4, ATP6V0A2, ZNF451, ALDH6A1, NKTR, BRWD1, PSMC2, PSMD10, NRIP1, LRCH3, RUNX3, SCRN1, ANKRD46
hsa‐miR‐1245	Down	14	CCR1, IFNAR1, NIT1, RHOT1, PTPN6, SLC2A11, SYK, TOP3A, LAT2, PPP1R3B, CRISPLD2, CFLAR, RASSF2, KIAA0232
hsa‐miR‐31*	Down	14	CAMKK2, CYP1B1, FKBP5, KCTD2, MYD88, SLC6A6, STAT3, TLE3, TRIM25, SNX27, KIAA0232, TXNIP, HSPA6, SP1
hsa‐miR‐609	Up	13	UST, CDK6, CTSC, DPY19L4, GRSF1, MAX, MGAT2, CREB3L2, ARV1, TRAM2, ALDH6A1, PLAC8, ACP1
hsa‐miR‐491‐3p	Down	10	FKBP5, NOV, PAK2, RHOT1, SLA, THBD, TPM3, SNX27, VAPA, SULT1B1
hsa‐miR‐155*	Down	9	IRAK3, FCHO2, RERE, NOTCH1, PITPNA, TRIM25, MRVI1, TXNIP, MYD88
hsa‐miR‐1468	Up	7	SEH1L, SLC30A7, ENPP4, ZZZ3, GNPTAB, CALM1, TMEM41A
hsa‐miR‐132*	Up	3	BCL2, RPL15, ACRBP
hsa‐miR‐487b	Down	1	MAP2K4

### Enrichment analyses of target genes of differentially expressed miRNA

**Figure 4:**
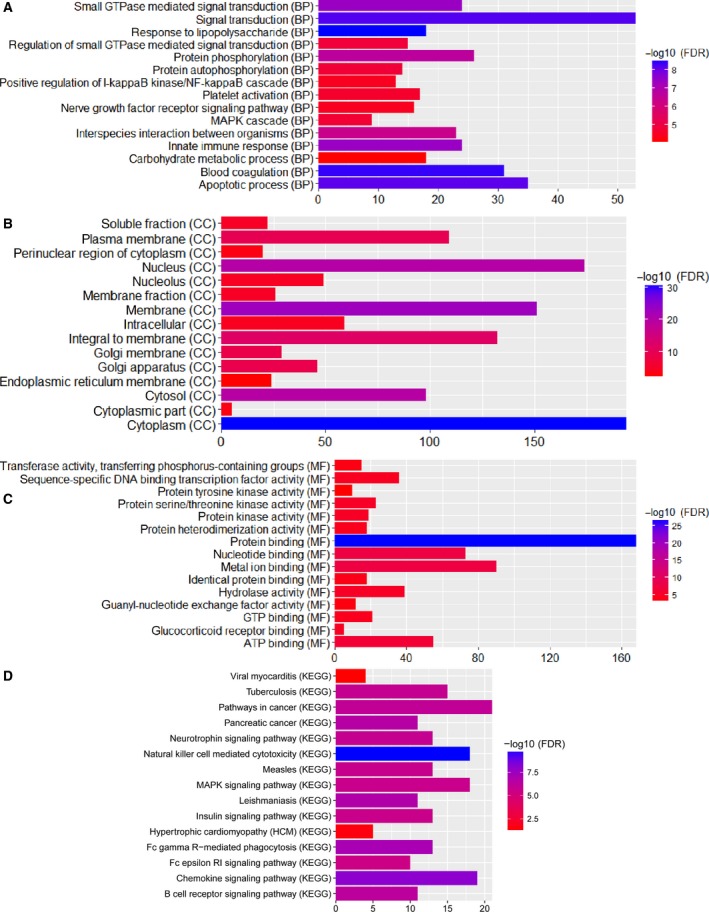
Top 15 significant enrichment GO and KEGG terms of DEGs. (A) BP: biological process; (B) CC: cellular component; (C) MF: molecular function; (D) KEGG: signaling pathway.

In target genes analysis of differentially expressed miRNA, a total of 528 target DEGs were obtained. To study the biological function of these target DEGs, GO enrichment and KEGG pathway analysis were performed. Based on the top 15 GO terms, enrichment analysis (Fig. 4), carbohydrate metabolic process, platelet activation, and nerve growth factor receptor signaling pathway were the most significantly enriched biological processes; intracellular, nucleolus, and membrane fraction were the most remarkably enriched cellular components; hydrolase activity, sequence‐specific DNA binding transcription factor activity, and protein serine/threonine kinase activity were the most significantly enriched molecular functions. Additionally, hypertrophic cardiomyopathy (HCM) and viral myocarditis were the most remarkably enriched signal pathways (Table 5 and Fig. 4).

**Table 5:** Enriched KEGG pathway of target DEGs in MI

Items	Items_Details	Count	Size	FDR	Symbols
04650	Natural killer cell‐mediated cytotoxicity	18	125	1.71E‐10	SH2D1B, MAPK3, PTK2B, RAC2, SH2D1A, IFNG, GRB2, SYK, HLA‐C, FASLG, PTPN6, PAK1, KLRD1, RAF1, PIK3CD, PPP3CA, IFNAR1, VAV3
04666	Fc gamma R‐mediated phagocytosis	13	92	8.05E‐08	MAPK3, WAS, RAC2, SYK, GAB2, DNM1L, FCGR2A, PAK1, RAF1, PIK3CD, INPP5D, VAV3, LIMK2
04662	B‐cell receptor signaling pathway	11	75	5.55E‐07	MAPK3, RAC2, GRB2, LILRB3, SYK, PTPN6, RAF1, PIK3CD, PPP3CA, INPP5D, VAV3
05200	Pathways in cancer	21	324	9.56E‐07	CDK6, STAT1, MAPK3, TGFA, MAX, RAC2, STAT3, TPM3, GRB2, RALB, CASP9, FASLG, RALBP1, SKP2, RAF1, PIK3CD, BCL2, ITGB1, RXRA, DAPK2, STAT5B
04722	Neurotrophin signaling pathway	13	124	1.41E‐06	YWHAH, MAPK3, YWHAQ, MAPKAPK2, GRB2, MAP3K3, FASLG, RAF1, IRAK3, PIK3CD, BCL2, RPS6KA1, IRS2
05152	Tuberculosis	15	172	1.50E‐06	STAT1, MAPK3, TLR2, ATP6V0A2, IFNG, SYK, CASP9, MYD88, FCGR2A, RAF1, BCL2, PPP3CA, NFYC, LAMP2, CR1
05162	Measles	13	130	2.02E‐06	CDK6, STAT1, TLR2, SH2D1A, STAT3, IFNG, HSPA6, MYD88, FASLG, PIK3CD, ADAR, STAT5B, IFNAR1
04010	MAPK signaling pathway	18	262	2.30E‐06	MAPK3, RASGRP4, MAX, IL1R1, RAC2, MAPKAPK2, GRB2, MAP3K3, MAP2K4, HSPA6, ELK4, FASLG, DUSP5, PAK1, RAF1, PPP3CA, RPS6KA1, PAK2
05410	HCM	5	82	0.0278085	ITGA4, TTN, TPM3, ITGB1, PRKAB1
05416	Viral myocarditis	4	63	0.043939	CD55, RAC2, CASP9, HLA‐C

### Protein–protein interaction network

**Figure 5:**
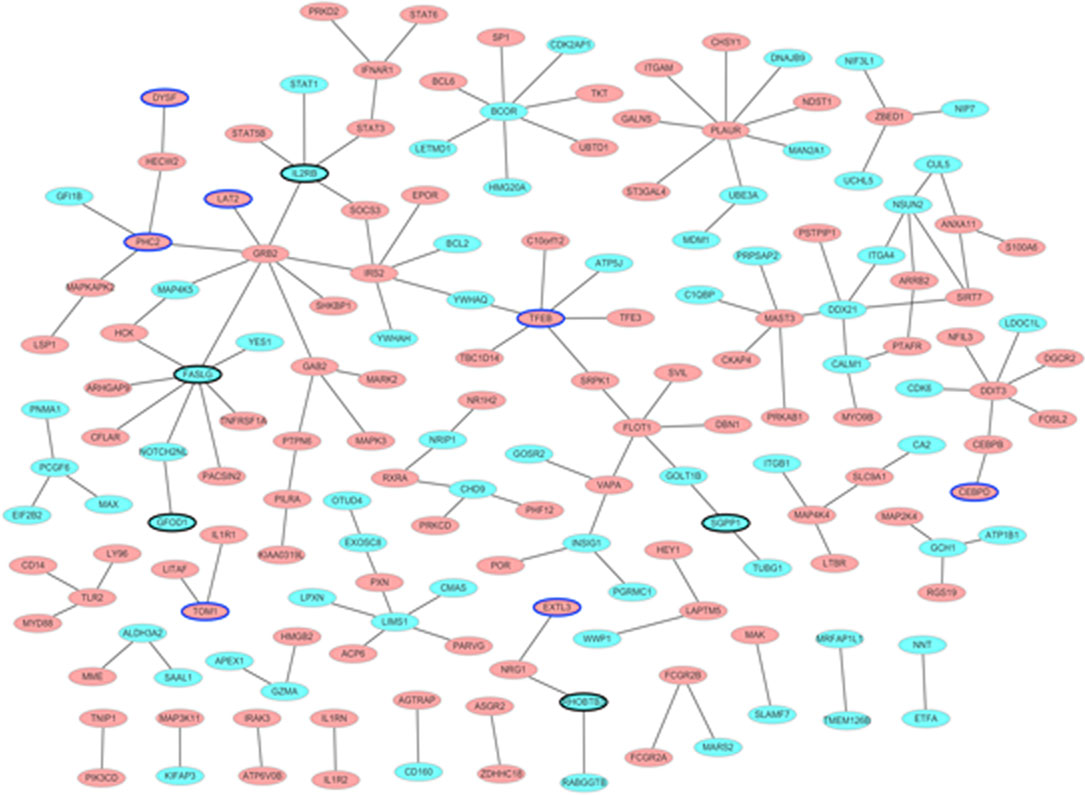
The PPI networks of top 100 DEGs. All the ellipses are proteins encoded by top 100 DEGs. The red and green colors represent up‐regulation and down‐regulation, respectively. Ellipses with blue and black border represent top 10 up‐regulation and down‐regulation, respectively.

To obtain the interaction between the proteins encoded by DEGs and other proteins, PPI network was explored and visualize by Cytoscape. PPI networks of top 50 up‐regulated and top 50 down‐regulated DEGs were shown in Fig. 5. As Fig. 5 shown, the network consisted of 170 nodes and 148 edges. The top twelve proteins with a high degree were GRB2 (degree = 8), PLAUR (degree = 8), FASLG (degree = 8), BCOR (degree = 7), TFEB (degree = 6), DDIT3 (degree = 6), IRS2 (degree = 6), MAST3 (degree = 5), DDX21 (degree = 5), IL2RB (degree = 5), LIMS1 (degree = 5), and FLOT1 (degree = 5).

### Receiver operating characteristic curve analysis

**Figure 6:**
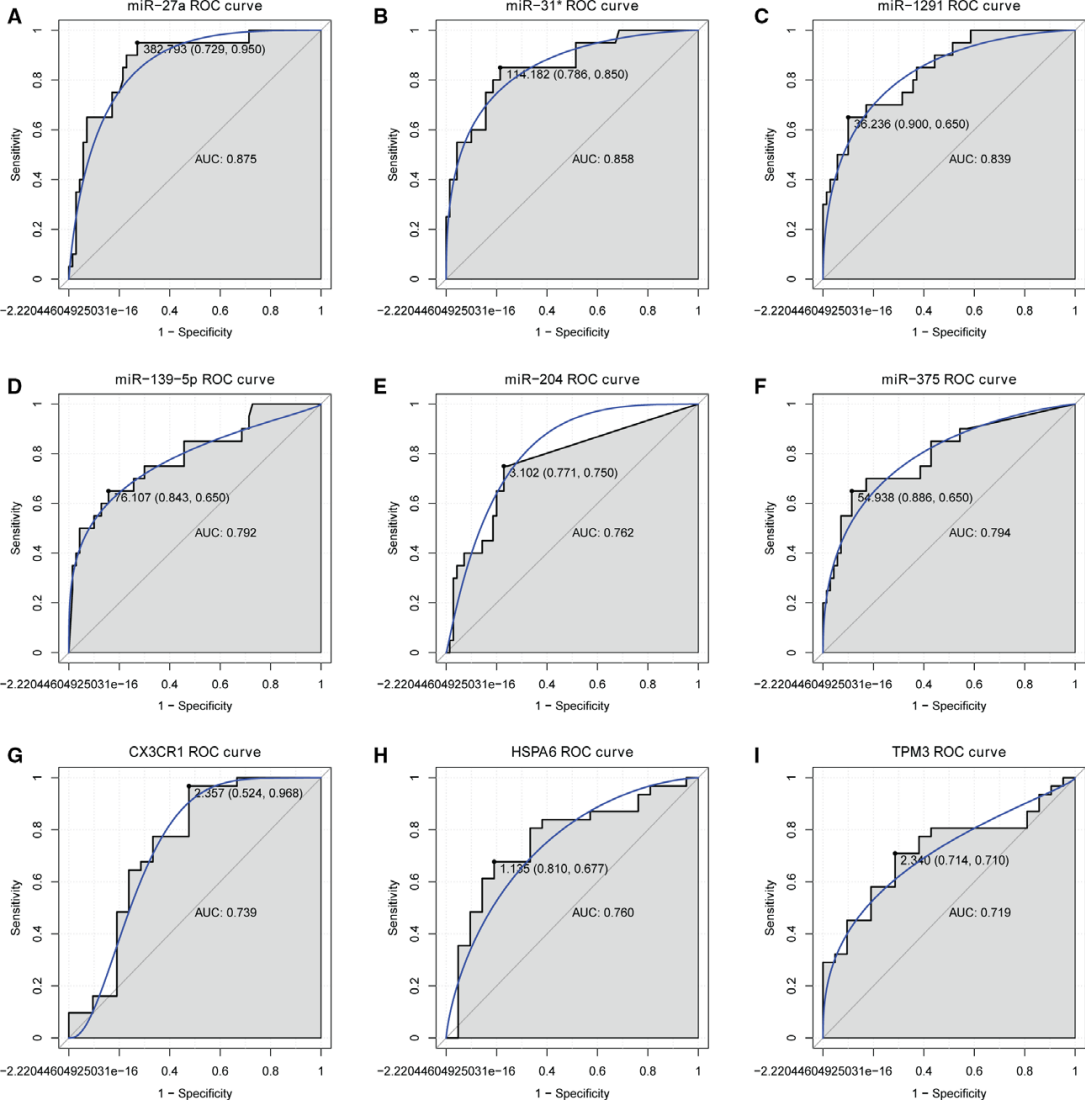
receiver operating characteristic curves of selected DEGs and differentially expressed miRNA between MI patients and healthy controls. (A) miR‐27a; (B) miR‐31*; (C) miR‐1291; (D) miR‐139‐5p; (E) miR‐204; (F) miR‐375; (G) CX3CR1; (H) HSPA6; (I) TPM3. The receiver operating characteristic curves were used to show the diagnostic ability of these selected DEGs and differentially expressed miRNA with specificity and sensitivity. The *x*‐axis shows 1‐specificity, and *y*‐axis shows sensitivity.

We performed receiver operating characteristic curve analyses and calculated the AUC to assess the discriminatory ability of selected six miRNA (miR‐27a, miR‐31*, miR‐139‐5p, miR‐204, miR‐375, and miR‐1291) and three DEGs (CX3CR1, HSPA6, and TPM3) from GEO dataset (Fig. 6). The AUC of all these DEGs and miRNA was > 0.7. MiR‐27a, miR‐31*, and miR‐1291 had the largest AUC. For MI diagnosis, the specificity and sensitivity of miR‐27a were 72.9% and 95%, respectively; the specificity and sensitivity of miR‐31* were 78.6% and 85%, respectively; the specificity and sensitivity of miR‐139‐5p were 84.3% and 65%, respectively; the specificity and sensitivity of miR‐204 were 77.1% and 75%, respectively; the specificity and sensitivity of miR‐375 were 88.6% and 65%, respectively; the specificity and sensitivity of miR‐1291 were 90% and 65%, respectively; the specificity and sensitivity of CX3CR1 were 96.8% and 52.4%, respectively; the specificity and sensitivity of HSPA6 were 67.7% and 81%, respectively; the specificity and sensitivity of TPM3 were 71% and 71.4%, respectively. In addition, the data of the receiver operating characteristic analysis including the C‐statistic and 95% confidence interval and odds ratio and 95% confidence interval were listed in Table 6.

**Table 6:** The data of receiver operating characteristic analysis

miRNA/mRNA	AUC	95% confidence interval	Odds ratio	95% confidence interval
miR‐27a	0.8754	0.7902–0.9605	51	6.3793–407.7280
miR‐31*	0.8575	0.7615–0.9535	20.7778	5.3666–80.4666
miR‐1291	0.8393	0.7432–0.9354	16.7143	5.0049–55.8192
miR‐139‐5p	0.7921	0.6707–0.9136	9.961	3.2439–30.5871
miR‐204	0.7625	0.6457–0.8793	10.125	3.1877–32.1598
miR‐375	0.7943	0.6725–0.9161	14.3929	4.4338–46.7221
CX3CR1	0.7389	0.5844–0.8933	33	3.7729–288.6339
HSPA6	0.7604	0.6221–0.8987	6.72	1.9153–23.5773
TPM3	0.7189	0.578–0.8598	6.1111	1.7972–20.7800

### Quantitative RT‐PCR

**Figure 7:**
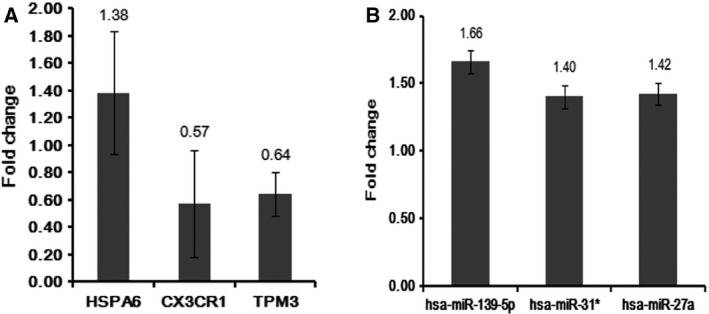
Validation differentially expressed miRNAs and genes in the MI blood by qRT‐PCR. (A) The expression of differentially expressed genes; (B) The expression of differentially expressed miRNAs.

To verify the bioinformatics analyses, the expression level of DEGs and differentially expressed miRNA was quantified by qRT‐PCR in five blood samples of MI patients and five normal blood samples. Three DEGs (HSPA6, CX3CR1, and TPM3) and three differentially expressed miRNA (miR‐139‐5p, miR‐31*, and miR‐27a) were selected for validation. As showed in Fig. 7, HSPA6, miR‐139‐5p, miR‐31*, and miR‐27a were up‐regulated and CX3CR1 and TPM3 were down‐regulated. The validation result was consistent with the bioinformatics except TPM3, miR‐139‐5p, and miR‐31*.

### Early stage expression analyses and recovery‐related analysis of differentially expressed genes

**Figure 8:**
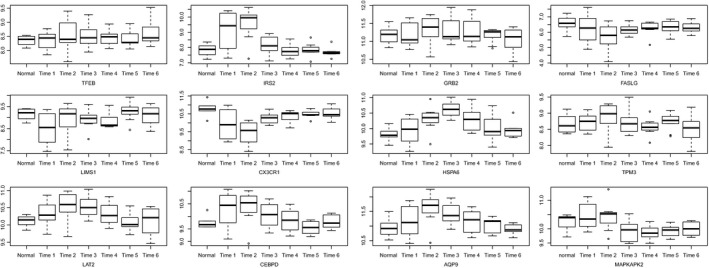
Expression of DEGs in early stage of MI. Time 1: 12 h after blood collection; Time 2: 24 h after blood collection; Time 3: 36 h after blood collection; Time 4: 72 h after blood collection; Time 5: 84 h after blood collection; Time 6: 96 h after blood collection.

**Figure 9:**
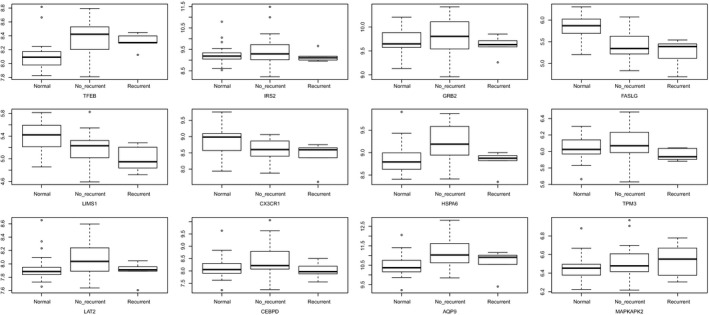
Recovery‐related analysis of DEGs.

As shown in Fig. 8, TFEB, IRS2, GRB2, FASLG, LIMS1, CX3CR1, HSPA6, TPM3, LAT2, CEBPD, AQP9, and MAPKAPK2 were differentially expressed in different time points. Furthermore, all these genes were associated with the recovery of MI (Fig. 9).

## Discussion

Myocardial infarction is an important clinical problem because of its large contribution to mortality. Therefore, it is urgent to elucidate MI carcinogenesis mechanism for developing novel diagnose that will be highly specific to malignant cells, with minimal or no risk of adverse effect. In this study, we found several differentially expressed miRNA and genes, which may play an important role in the development of MI.

Linker for activation of T cells family member 2 is involved in the process of calcium mobilization, which is associated with coronary artery calcification in atherosclerosis 27. Herein, we found that LAT2 was regulated by both miR‐767‐5p and miR‐1245 in the blood of MI. MiR‐767‐5p has been found differentially expressed in the heart tissue of patients with MI 11. However, there are not any reports about miR‐1245 in the development of heart. Further research is needed to study the function of miR‐1245.

CCAAT/enhancer binding protein delta (also called CELF) is a transcription factor important in activating the expression of inflammatory genes in cardiac myocytes 28. In animal models, the expression of CEBPD altered in skeletal and muscular functions and overexpression of the dominant negative CEBPD protein results in fibrosis, cardiac hypertrophy, and dilated cardiomyopathy 29, 30, 31. In our study, CEBPD was regulated by miR‐455‐3p in the blood of MI. It is noted that the expression of miR‐455‐3p was deregulated early during acute MI 32.

C‐X3‐C motif chemokine receptor 1 is associated with the prevalence of coronary heart disease or MI 33. In this study, we found that CX3CR1 was under the regulation of miR‐27a. Romaine *et al*. 34 found that miR‐27a had a high specificity in predicting the occurrence of left ventricular failure 6 months after acute MI. It is worth mentioning that miR‐27a is the prognostic indicator for acute MI 35. In this study, we found that both miR‐27a and target gene CX3CR1 had a great diagnostic value for MI.

Aquaporin 9 is a gap junction network gene that is vital to heart function. It is reported that AQP9 is also related to acute MI 36. Kuiper *et al*. 37 found that TFEB was expressed in the myocardium of the adult. In this study, we found that AQP9 and TFEB were under the regulation of miR‐330‐3p in blood of MI. It is noted that miR‐330‐3p is up‐regulated in heart but down‐regulated in the plasma of patients with chronic heart failure 38.

In addition, we also found several DEGs (such as IRS2, GRB2, FASLG, and LIMS1) with a high degree in the PPI network. IRS2 plays a key role in cardiac homeostasis regulation 39. Zawada *et al*. 40 also found that GRB2 plays an important role in the signaling pathway for cardiac hypertrophy. Herein, we found both IRS2 and GRB2 were regulated by miR‐200a. It is demonstrated that miR‐200a is involved in the cardiovascular differentiation 41.

Fas ligand (also called TNFSF6) is a member of the TNF family and the main activator of the extrinsic apoptotic pathway that binds the TNF receptor to induce apoptosis during MI 42. LIMS1 (also called PINCH1) has been suggested to be associated with left‐sided congenital heart disease 43. In this study, FASLG and LIMS1 were under the regulation of miR‐520c‐3p. It is found that treatment with miR‐520c will increase MMP‐9 expression, which regulates remodeling of the left ventricle after MI and is tightly linked to the inflammatory response 44.

It is reported that MAPK is a key signal pathway in MI 45. According to KEGG pathway enrichment analysis in MI, MAPK signal pathway was found covered the most DEGs, such as HSPA6 and MAPKAPK2. HSPA6 is found to be a regulated protein in planned MI patient samples 46. MAPKAPK2 is the substrate for p38‐MAPK and less abundant in failing heart 47, 48. In the present study, we found that HSPA6 and MAPKAPK2 were regulated by miR‐31* and miR‐1291, respectively. It is worth mentioning that HSPA6 and miR‐31* had a great diagnose value for MI. MiR‐1291 has been identified as a potential diagnosis biomarker for acute MI 49.

Clinically, HCM is defined in the presence of left ventricular hypertrophy in the absence of hypertension and valve disease. In this study, HCM was found to be the most enriched signal pathway, which involved several genes such as TPM3. TPM3 is found to be a regulated protein in planned MI patient samples 46. Herein, we found that TPM3 was regulated by miR‐139‐5p, and both miR‐139‐5p and TPM3 had a great diagnose value for MI. In human autopsy samples, miR‐139‐5p is down‐regulated earlier, within just 7 days following MI 11.

Beside miR‐27a, miR‐31*, and miR‐139‐5p, we also found that miR‐204 and miR‐375 had the diagnose value for MI. MiR‐204 is an autophagy‐modulating miRNA that was related to cardiovascular disease 50. It is reported that the expression of miR‐375 is remarkably up‐regulated in heart tissue of MI and circulating miR‐375 is a potential diagnostic biomarker for MI 51, 52. In this study, we found both miR‐204 and miR‐375 had a great diagnose value for MI.

To analyze the expression of TFEB, IRS2, GRB2, FASLG, LIMS1, CX3CR1, HSPA6, TPM3, LAT2, CEBPD, AQP9, and MAPKAPK2 in the early stage of MI and further study the association between these genes with MI recovery, the datasets of http://www.ncbi.nlm.nih.gov/geo/query/acc.cgi?acc=GSE29532 and http://www.ncbi.nlm.nih.gov/geo/query/acc.cgi?acc=GSE48060 were used for analysis. Our results showed that all these genes were differentially expressed in different blood collection points. Moreover, these genes were associated with the recovery of MI. Therefore, we inferred that these genes may be considered as biomarkers for early stages of MI, as well as for monitoring early MI recovery.

In summary, we found several differentially expressed miRNA and genes in the blood of MI. MiR‐27a, miR‐31*, miR‐1291, miR‐139‐5p, miR‐204, miR‐375, and target genes including CX3CR1, HSPA6, and TPM3 had a great diagnose value for MI. Additionally, MAPK and HCM were important signal pathways in the development of MI. TFEB, IRS2, GRB2, FASLG, LIMS1, CX3CR1, HSPA6, TPM3, LAT2, CEBPD, AQP9, and MAPKAPK2 may regard as biomarkers in MI early stage and recovery. Our study may be helpful in understanding the pathology mechanism of MI and could provide the clues in clinical diagnose and drug design of MI. There are limitations to our study. Firstly, sample size in the qRT‐PCR was small. Larger numbers of blood samples are needed for further research. Secondly, some animal models and cell culture experiments are needed to validate and explore the potential function of identified differentially expressed miRNA and genes in MI.

## Author contributions

QuZ conceived and supervised the study; QuZ, KW, and QiZ designed experiments; ZL and NL performed experiments; QX and XL analyzed data; KW and QiZ wrote the manuscript; KW, QiZ, and QuZ made manuscript revisions.

## Supporting information


**Table S1**. All differentially expressed genes.

## References

[1] Rodriguez M , Cai WJ , Kostin S , Lucchesi BR and Schaper J (2005) Ischemia depletes dystrophin and inhibits protein synthesis in the canine heart: mechanisms of myocardial ischemic injury. J Mol Cell Cardiol 38, 723–733. 15850566 10.1016/j.yjmcc.2005.02.019

[2] Yamada Y , Ichihara S and Nishida T (2008) Molecular genetics of myocardial infarction. Genom Med 2, 7–22. 10.1007/s11568-008-9025-x PMC2518661 18704761

[3] Swirski FK and Nahrendorf M (2013) Leukocyte behavior in atherosclerosis, myocardial infarction, and heart failure. Science 339, 161–166. 23307733 10.1126/science.1230719 PMC3891792

[4] Weinberger T and Schulz C (2015) Myocardial infarction: a critical role of macrophages in cardiac remodeling. Front Physiol 6, 107. 25904868 10.3389/fphys.2015.00107 PMC4387471

[5] Goldberger JJ , Bonow RO , Cuffe M , Liu L , Rosenberg Y , Shah PK , Smith SC Jr and Subacius H (2015) Effect of beta‐blocker dose on survival after acute myocardial infarction. J Am Coll Cardiol 66, 1431–1441. 26403339 10.1016/j.jacc.2015.07.047 PMC4583654

[6] Liu N and Olson EN (2010) MicroRNA regulatory networks in cardiovascular development. Dev Cell 18, 510–525. 20412767 10.1016/j.devcel.2010.03.010 PMC2922691

[7] Vickers KC , Rye KA and Tabet F (2014) MicroRNAs in the onset and development of cardiovascular disease. Clin Sci (Lond) 126, 183–194. 24102098 10.1042/CS20130203 PMC3873876

[8] Porrello ER (2013) microRNAs in cardiac development and regeneration. Clin Sci (Lond) 125, 151–166. 23634935 10.1042/CS20130011

[9] Wen Z , Zheng S , Zhou C , Yuan W , Wang J and Wang T (2012) Bone marrow mesenchymal stem cells for post‐myocardial infarction cardiac repair: microRNAs as novel regulators. J Cell Mol Med 16, 657–671. 22004043 10.1111/j.1582-4934.2011.01471.x PMC3822837

[10] Meder B , Keller A , Vogel B , Haas J , Sedaghat‐Hamedani F , Kayvanpour E , Just S , Borries A , Rudloff J , Leidinger P *et al* (2011) MicroRNA signatures in total peripheral blood as novel biomarkers for acute myocardial infarction. Basic Res Cardiol 106, 13–23. 20886220 10.1007/s00395-010-0123-2

[11] Bostjancic E , Zidar N and Glavac D (2009) MicroRNA microarray expression profiling in human myocardial infarction. Dis Markers 27, 255–268. 20075508 10.3233/DMA-2009-0671 PMC3834671

[12] Liang H , Zhang C , Ban T , Liu Y , Mei L , Piao X , Zhao D , Lu Y , Chu W and Yang B (2012) A novel reciprocal loop between microRNA‐21 and TGFbetaRIII is involved in cardiac fibrosis. Int J Biochem Cell Biol 44, 2152–2160. 22960625 10.1016/j.biocel.2012.08.019

[13] Shan ZX , Lin QX , Fu YH , Deng CY , Zhou ZL , Zhu JN , Liu XY , Zhang YY , Li Y , Lin SG *et al* (2009) Upregulated expression of miR‐1/miR‐206 in a rat model of myocardial infarction. Biochem Biophys Res Commun 381, 597–601. 19245789 10.1016/j.bbrc.2009.02.097

[14] Shi B , Guo Y , Wang J and Gao W (2010) Altered expression of microRNAs in the myocardium of rats with acute myocardial infarction. BMC Cardiovasc Disord 10, 11. 20187981 10.1186/1471-2261-10-11 PMC2844352

[15] Deleon‐Pennell KY , Altara R , Yabluchanskiy A , Modesti A , Lindsey ML (2015) The circular relationship between matrix metalloproteinase‐9 and inflammation following myocardial infarction. IUBMB Life 67, 611–618. 26269290 10.1002/iub.1408 PMC4553095

[16] Alpert JS , Thygesen K , Antman E and Bassand JP (2000) Myocardial infarction redefined – A consensus document of The Joint European Society of Cardiology/American College of Cardiology Committee for the Redefinition of Myocardial Infarction Eur Heart J 2000 21 1502 1513 10973764. J Am Coll Cardiol 36, 959–969. 10987628 10.1016/s0735-1097(00)00804-4

[17] Luchner A , Hengstenberg C , Lowel H , Trawinski J , Baumann M , Riegger GA , Schunkert H and Holmer S (2002) N‐terminal pro‐brain natriuretic peptide after myocardial infarction: a marker of cardio‐renal function. Hypertension 39, 99–104. 11799086 10.1161/hy0102.100537

[18] de Winter RJ , Koster RW , Sturk A and Sanders GT (1995) Value of myoglobin, troponin T, and CK‐MBmass in ruling out an acute myocardial infarction in the emergency room. Circulation 92, 3401–3407. 8521560 10.1161/01.cir.92.12.3401

[19] Wang GK , Zhu JQ , Zhang JT , Li Q , Li Y , He J , Qin YW and Jing Q (2010) Circulating microRNA: a novel potential biomarker for early diagnosis of acute myocardial infarction in humans. Eur Heart J 31, 659–666. 20159880 10.1093/eurheartj/ehq013

[20] Long G , Wang F , Duan Q , Chen F , Yang S , Gong W , Wang Y , Chen C and Wang DW (2012) Human circulating microRNA‐1 and microRNA‐126 as potential novel indicators for acute myocardial infarction. Int J Biol Sci 8, 811–818. 22719221 10.7150/ijbs.4439 PMC3372885

[21] Yao XL , Lu XL , Yan CY , Wan QL , Cheng GC and Li YM (2015) Circulating miR‐122‐5p as a potential novel biomarker for diagnosis of acute myocardial infarction. Int J Clin Exp Pathol 8, 16014–16019. 26884877 PMC4730090

[22] Zhong J , He Y , Chen W , Shui X , Chen C and Lei W (2014) Circulating microRNA‐19a as a potential novel biomarker for diagnosis of acute myocardial infarction. Int J Mol Sci 15, 20355–20364. 25383678 10.3390/ijms151120355 PMC4264171

[23] Marot G , Foulley JL , Mayer CD and Jaffrezic F (2009) Moderated effect size and P‐value combinations for microarray meta‐analyses. Bioinformatics 25, 2692–2699. 19628502 10.1093/bioinformatics/btp444

[24] Reiner‐Benaim A (2007) FDR control by the BH procedure for two‐sided correlated tests with implications to gene expression data analysis. Biom J 49, 107–126. 17342953 10.1002/bimj.200510313

[25] Benjamini Y and Hochberg Y (1995) Controlling the false discovery rate ‐ a practical and powerful approach to multiple testing. J Roy Stat Soc 57, 289–300.

[26] Smoot ME , Ono K , Ruscheinski J , Wang PL and Ideker T (2011) Cytoscape 2.8: new features for data integration and network visualization. Bioinformatics 27, 431–432. 21149340 10.1093/bioinformatics/btq675 PMC3031041

[27] Huang CC , Lloyd‐Jones DM , Guo X , Rajamannan NM , Lin S , Du P , Huang Q , Hou L and Liu K (2011) Gene expression variation between African Americans and whites is associated with coronary artery calcification: the multiethnic study of atherosclerosis. Physiol Genomics 43, 836–843. 21521779 10.1152/physiolgenomics.00243.2010 PMC3132836

[28] Zgheib C , Kurdi M , Zouein FA , Gunter BW , Stanley BA , Zgheib J , Romero DG , King SB , Paolocci N and Booz GW (2012) Acyloxy nitroso compounds inhibit LIF signaling in endothelial cells and cardiac myocytes: evidence that STAT3 signaling is redox‐sensitive. PLoS ONE 7, e43313. 22905257 10.1371/journal.pone.0043313 PMC3419695

[29] Galindo CL , Skinner MA , Errami M , Olson LD , Watson DA , Li J , McCormick JF , McIver LJ , Kumar NM , Pham TQ *et al* (2009) Transcriptional profile of isoproterenol‐induced cardiomyopathy and comparison to exercise‐induced cardiac hypertrophy and human cardiac failure. BMC Physiol 9, 23. 20003209 10.1186/1472-6793-9-23 PMC2799380

[30] Ladd AN , Taffet G , Hartley C , Kearney DL and Cooper TA (2005) Cardiac tissue‐specific repression of CELF activity disrupts alternative splicing and causes cardiomyopathy. Mol Cell Biol 25, 6267–6278. 15988035 10.1128/MCB.25.14.6267-6278.2005 PMC1168813

[31] Wang GS , Kearney DL , De Biasi M , Taffet G and Cooper TA (2007) Elevation of RNA‐binding protein CUGBP1 is an early event in an inducible heart‐specific mouse model of myotonic dystrophy. J Clin Invest 117, 2802–2811. 17823658 10.1172/JCI32308 PMC1964514

[32] Schulte C and Zeller T (2015) microRNA‐based diagnostics and therapy in cardiovascular disease‐Summing up the facts. Cardiovasc Diagn Ther 5, 17–36. 25774345 10.3978/j.issn.2223-3652.2014.12.03 PMC4329169

[33] Lavergne E , Labreuche J , Daoudi M , Debre P , Cambien F , Deterre P , Amarenco P and Combadiere C (2005) Adverse associations between CX3CR1 polymorphisms and risk of cardiovascular or cerebrovascular disease. Arterioscler Thromb Vasc Biol 25, 847–853. 15681302 10.1161/01.ATV.0000157150.23641.36

[34] Romaine SP , Tomaszewski M , Condorelli G and Samani NJ (2015) MicroRNAs in cardiovascular disease: an introduction for clinicians. Heart 101, 921–928. 25814653 10.1136/heartjnl-2013-305402 PMC4484262

[35] Devaux Y , Vausort M , McCann GP , Kelly D , Collignon O , Ng LL , Wagner DR and Squire IB (2013) A panel of 4 microRNAs facilitates the prediction of left ventricular contractility after acute myocardial infarction. PLoS ONE 8, e70644. 23967079 10.1371/journal.pone.0070644 PMC3742776

[36] Maciejak A , Kiliszek M , Michalak M , Tulacz D , Opolski G , Matlak K , Dobrzycki S , Segiet A , Gora M and Burzynska B (2015) Gene expression profiling reveals potential prognostic biomarkers associated with the progression of heart failure. Genome Med 7, 26. 25984239 10.1186/s13073-015-0149-z PMC4432772

[37] Kuiper RP , Schepens M , Thijssen J , Schoenmakers EF and van Kessel AG (2004) Regulation of the MiTF/TFE bHLH‐LZ transcription factors through restricted spatial expression and alternative splicing of functional domains. Nucleic Acids Res 32, 2315–2322. 15118077 10.1093/nar/gkh571 PMC419459

[38] Li H , Fan J , Yin Z , Wang F , Chen C and Wang DW (2016) Identification of cardiac‐related circulating microRNA profile in human chronic heart failure. Oncotarget 7, 33–45. 26683101 10.18632/oncotarget.6631 PMC4807981

[39] Qi Y , Xu Z , Zhu Q , Thomas C , Kumar R , Feng H , Dostal DE , White MF , Baker KM and Guo S (2013) Myocardial loss of IRS1 and IRS2 causes heart failure and is controlled by p38alpha MAPK during insulin resistance. Diabetes 62, 3887–3900. 24159000 10.2337/db13-0095 PMC3806607

[40] Zawada AM , Rogacev KS , Hummel B , Grün OS , Friedrich A , Rotter B , Winter P , Geisel J , Fliser D and Heine GH (2012) SuperTAG methylation‐specific digital karyotyping reveals uremia‐induced epigenetic dysregulation of atherosclerosis‐related genes. Cir Cardiovasc Genet 5, 611. 10.1161/CIRCGENETICS.112.963207 23074332

[41] Zaccagnini G , Martelli F , Fasanaro P , Magenta A , Gaetano C , Di Carlo A , Biglioli P , Giorgio M , Martin‐Padura I , Pelicci PG *et al* (2004) p66ShcA modulates tissue response to hindlimb ischemia. Circulation 109, 2917–2923. 15173034 10.1161/01.CIR.0000129309.58874.0F

[42] Lee P , Sata M , Lefer DJ , Factor SM , Walsh K and Kitsis RN (2003) Fas pathway is a critical mediator of cardiac myocyte death and MI during ischemia‐reperfusion *in vivo* . Am J Physiol Heart Circ Physiol 284, H456–H463. 12414449 10.1152/ajpheart.00777.2002

[43] Hitz MP , Lemieux‐Perreault LP , Marshall C , Feroz‐Zada Y , Davies R , Yang SW , Lionel AC , D'Amours G , Lemyre E , Cullum R *et al* (2012) Rare copy number variants contribute to congenital left‐sided heart disease. PLoS Genet 8, e1002903. 22969434 10.1371/journal.pgen.1002903 PMC3435243

[44] Deleon‐Pennell KY , Altara R , Yabluchanskiy A , Modesti A and Lindsey ML (2015) The circular relationship between matrix metalloproteinase‐9 and inflammation following myocardial infarction. IUBMB Life 67, 611–618. 26269290 10.1002/iub.1408 PMC4553095

[45] Wang P , Haiying FU , Cui J and Chen X (2016) Differential lncRNA‐mRNA co‐expression network analysis revealing the potential regulatory roles of lncRNAs in myocardial infarction. Mol Med Rep 13, 1195. 26676325 10.3892/mmr.2015.4669 PMC4732855

[46] Keshishian H , Burgess MW , Gillette MA , Mertins P , Clauser KR , Mani DR , Kuhn EW , Farrell LA , Gerszten RE and Carr SA (2015) Multiplexed, quantitative workflow for sensitive biomarker discovery in plasma yields novel candidates for early myocardial injury. Mol Cell Proteomics 14, 2375–2393. 25724909 10.1074/mcp.M114.046813 PMC4563722

[47] Rouse J , Cohen P , Trigon S , Morange M , Alonso‐Llamazares A , Zamanillo D , Hunt T and Nebreda AR (1994) A novel kinase cascade triggered by stress and heat shock that stimulates MAPKAP kinase‐2 and phosphorylation of the small heat shock proteins. Cell 78, 1027–1037. 7923353 10.1016/0092-8674(94)90277-1

[48] Kotlo K , Johnson KR , Grillon JM , Geenen DL , deTombe P and Danziger RS (2012) Phosphoprotein abundance changes in hypertensive cardiac remodeling. J Proteomics 77, 1–13. 22659219 10.1016/j.jprot.2012.05.041 PMC3581302

[49] Peng L , Chun‐guang Q , Bei‐fang L , Xue‐zhi D , Zi‐hao W , Yun‐fu L , Yan‐ping D , Yang‐gui L , Wei‐guo L , Tian‐yong H *et al* (2014) Clinical impact of circulating miR‐133, miR‐1291 and miR‐663b in plasma of patients with acute myocardial infarction. Diagn Pathol 9, 89. 24885383 10.1186/1746-1596-9-89 PMC4082297

[50] Skommer J , Rana I , Marques FZ , Zhu W , Du Z and Charchar FJ (2014) Small molecules, big effects: the role of microRNAs in regulation of cardiomyocyte death. Cell Death Dis 5, e1325. 25032848 10.1038/cddis.2014.287 PMC4123081

[51] Garikipati VN , Krishnamurthy P , Verma SK , Khan M , Abramova T , Mackie AR , Qin G , Benedict C , Nickoloff E , Johnson J *et al* (2015) Negative regulation of miR‐375 by interleukin‐10 enhances bone marrow‐derived progenitor cell‐mediated myocardial repair and function after myocardial infarction. Stem Cells 33, 3519–3529. 26235810 10.1002/stem.2121 PMC4713300

[52] D'Alessandra Y , Devanna P , Limana F , Straino S , Di Carlo A , Brambilla PG , Rubino M , Carena MC , Spazzafumo L , De Simone M *et al* (2010) Circulating microRNAs are new and sensitive biomarkers of myocardial infarction. Eur Heart J 31, 2765–2773. 20534597 10.1093/eurheartj/ehq167 PMC2980809

